# Phytochemicals and Biological Activities of *Garcinia atroviridis*: A Critical Review

**DOI:** 10.3390/toxics10110656

**Published:** 2022-10-29

**Authors:** Muhammad Shahid, Douglas Law, Ahmad Azfaralariff, Mukram M. Mackeen, Teek Foh Chong, Shazrul Fazry

**Affiliations:** 1 Department of Biological Sciences and Biotechnology, Faculty of Science and Technology, Universiti Kebangsaan Malaysia, Bangi 43600, Malaysia; 2 Faculty of Health and Life Sciences, Inti International University, Persiaran Perdana BBN Putra Nilai, Nilai 71800, Malaysia; 3 Green Biopolymer, Coating and Packaging Cluster, School of Industrial Technology, Universiti Sains Malaysia, Gelugor 11800, Malaysia; 4 Department of Chemical Sciences, Faculty of Science and Technology, Universiti Kebangsaan Malaysia, Bangi 43600, Malaysia; 5 Institute of Systems Biology (INBIOSIS), Universiti Kebangsaan Malaysia, Bangi 43600, Malaysia; 6 Department of Food Sciences, Faculty of Science and Technology, Universiti Kebangsaan Malaysia, Bangi 43600, Malaysia

**Keywords:** *Garcinia atriviridis*, therapeutic potential, chemical constituent

## Abstract

*Garcinia atriviridis* Griff ex T. Anders (*G. atroviridis*) is one of the well-known species of the genus *Garicinia* that is native to Thailand, Myanmar, Peninsular Malaysia, and India. *G. atroviridis* is a perennial medium-sized tree that has a wide range of values, from food to medicinal use. Different parts of *G. atroviridis* are a great source of bioactive substances that have a positive impact on health. The extracts or bioactive constituents from *G. atroviridis* have demonstrated various therapeutic functions, including antioxidant, antimicrobial, anticancer, anti-inflammatory, antihyperlipidemic, and anti-diabetic. In this paper, we provide a critical review of *G. atroviridis* and its bioactive constituents in the prevention and treatment of different diseases, which will provide new insight to explore its putative domains of research.

## 1. Introduction

Natural herbal products (NHP) have a long history since they are widely used to treat various diseases. In every region of the world, independent plant-based therapeutic modalities have evolved throughout history, including Ayurveda in India, Sa-Sang in Korea, Kampo in Japan, and Traditional Chinese Medicine (TCM) [1]. In the nineteenth century, the discovery of a pure compound (morphine) replaced the NHPs. However, NHPs lost their importance as a result of the rapid progress in the field of chemistry in the 20th and 21st centuries, which encouraged high-throughput screening of synthetic chemical libraries for drug discovery [2]. In the last few decades, chemicals derived from NHPs have gained momentum because of their effectiveness, lower adverse effects, and better compatibility with the human body [3]. NHPs and their preparations play a significant role in animal medicine, food, cosmetics, and many other fields. The utilization of NHPs is advantageous because they are primarily derived from renewable resources and provide the origin of stereochemistry and optical activity of compounds [4].

*Garcinia atriviridis* Griff ex T. Anders. (*G. atroviridis*) is native and extensively distributed in Thailand, Myanmar, Peninsular Malaysia, and India. It belongs to the tropical family Guttiferae, often known as the Clusiaceae, which consists of 40 genera and over 1000 species. In Malaysia, only four genera, namely, *Garcinia*, *Calophyllum*, *Mesua*, and *Mammea*, are found in various habitats. *Garcinia* is a commercially important Guttiferae genus with over 400 species found in palaeotropical climates, primarily in Southeast Asia [5,6]. The genus species are mainly small to medium-sized dioecious evergreen fruit trees, occasionally shrubs, with hard wood and abundant latex. *Garcinia atriviridis* has different native names in each of these countries. The English or common name is “Asam Gelugor”, or “Som-Khaek” [7]. Local native names in Indonesia are: asam gelugor, asam potong; in Thailand: a sa ka lu ko (Malay), som-khaai, ma-khaam khaek, cha muang chang, som-pha-ngum, som-khaek, som-ma-won; and in Malaysia: assam gelugor, boh no, nayo (Semang), asam gelugo, and asam keping [5,7]. The sun-dried fruits of agelugor are cut into thin slices, which are known as “asam keeping” and are sold commercially in this region. These “Asam Kepings” are a popular spice for curries, sour relishes, and fish dressings [8]. The dried fruit slices are used to provide acidity to cooked foods. Nowadays, som-khaek products are becoming increasingly popular as health foods in Thailand, and a variety of som-khaek goods, such as som-khaek capsules, fruit slices, and tea, are available in markets. In Malaysia, the young, tender green shoots and leaves are consumed raw or cooked as ulam, or a sour relish. Garcinia plant parts have medicinal properties in addition to being used in food products [5]. Currently, the extracts or bioactive constituents from *G. atroviridis* demonstrate a broad range of biological functions, including antioxidant, antimicrobial, anticancer, anti-inflammatory, antihyperlipidemic, and anti-diabetic. Due to these reported therapeutic effects, this plant has gained the attention of several research groups over the last decade. According to the researchers, its distinct flavor and spiciness will enhance its popularity worldwide, as well as it being low in fat and calorie content and having medicinal values. In this paper, we provide a critical review of *G. atroviridis* and its bioactive constituents in the prevention and treatment of different diseases which will provide a new insight to explore its putative domains of research.

## 2. Geographical Distribution

**Figure 1:**
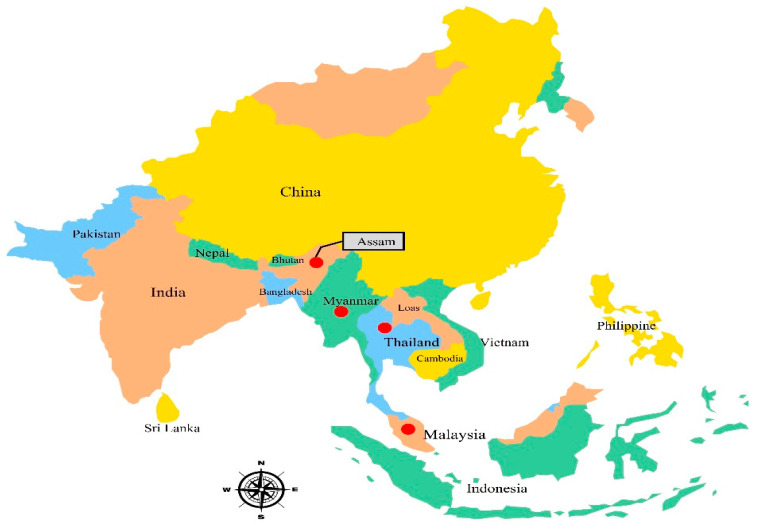
Geographical distribution of *G. atroviridis.* Red spots represent the distribution of *G. atroviridis*.

In Peninsular Malaysia, *Garcinia* is the largest genus of Guttiferae, and its fruit trees, especially *G. atroviridis*, *G. dulcis*, *G. prainiana*, *G. mangostana*, and *G. cowa*, are popular locally [9]. The members of this species grow individually and can be found in humid, mixed lowland forests and up to 600 meters above sea level in the highlands of high rainfall areas in Southeast Asia [5]. In Peninsular Malaysia, there are around 49 species of *Garcinia*; however, the identification of some of them, particularly the group of high mountain species, is still incomplete. This tree is native and extensively distributed in Thailand, Myanmar, Peninsular Malaysia, and India (Assam) (Figure 1). It is broadly cultivated in southern Thailand and Myanmar [5].

## 3. Botanical Description

**Figure 2:**
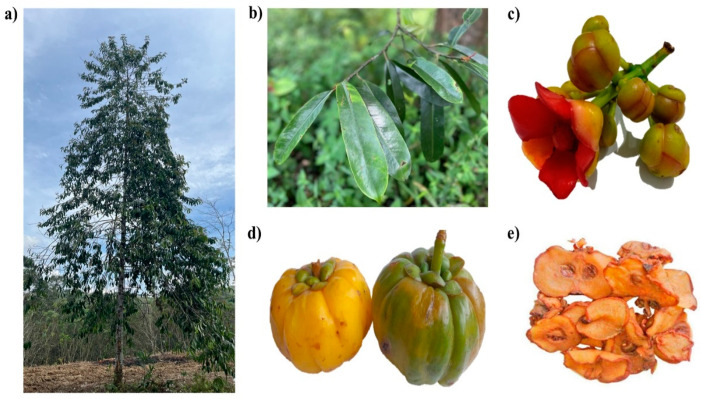
Different parts of *G. atroviridis*. (**a**) The *G. atroviridis* tree, (**b**) leaves, (**c**) flower, (**d**) ripened (yellow) and non-ripened (green) fruits, and (**e**) dried fruit slices (Asam Keping) from *G. atroviridis*.

*Garcinia atroviridis* is a perennial and medium-sized tree with a deep monopodial crown of thick, slender, drooping branches that grows up to 27 m in height and 70 cm in girth [10]. The glossy-leathery greenish-black leaves, tapering at the apex and base, are large and oblong-shaped, ranging between 15 × 4 and 25 × 7 cm on 15–25 mm long petioles [5,9]. The young leaves are occasionally used in cooking and as a traditional vegetable (ulam). The flowers terminate at the twigs and have four yellow concave sepals and four crimson fleshy petals. Male flowers have a fleshy receptacle that exhibits a bunch of stamens in whorls that round the pistillode [9]. Female flowers are solitary and have a large ovoid ribbed, 8–16 celled ovary. Its stigma is convex-shaped, and staminodes are attached to an annulus. Fruits have large, persistent petals and sepals, flattened seeds contained in the sour pink-white flesh (arillode), and a thick rind [9]. These are green, turning to brilliant yellow upon ripening, and reaching up to 10 cm in diameter; a fully ripe fruit can weigh up to 2 kg (Figure 2) [5].

## 4. Traditional Uses

Ethnopharmacological reports have documented the importance of some species that are used in Malayan folklore medicine [11]. Among them, two species, *G. mangostana* and *G. atroviridis*, have important therapeutic characteristics. The species *G. mangostana* is renowned for the effectiveness of its bark and dried rind in treating diarrhea and dysentery in Southeast Asia and India [12]. On the other hand, reports on the usage of *G. atroviridis* for medical reasons have been limited to Peninsular Malaysia and Sumatra. An infusion of *G. atroviridis* and *Ananas comosus* leaves is consumed in Sumatra to cure stomach discomfort caused by pregnancy [13]. In Southeast Asian traditional applications, *G. atroviridis* is utilized as a postpartum medication as well as a treatment for cough, throat irritation, earache, dandruff, and any stomach ache related to pregnancy [11]. In Thailand, som-khaek is used for boosting blood circulation, acting as an expectorant, treating coughs, and relieving constipation [5]. In Peninsular Malaysia, the fruit is used in a lotion with vinegar to apply on a woman’s belly after confinement. Juice from the leaves is administered to a woman after childbirth [11].

## 5. Proximate Composition and Phytochemical Reports of *G. atroviridis*

While *G. atroviridis* is a native of Peninsular Malaysia, Thailand, Myanmar, and India, information about this plant is still developing. The information on the proximate composition of this plant is still limited and not well reported. Table 1 lists the proximate composition of the plant that has been reported.

Lim (2012) has reported the proximate composition of the leaf and dried fruit, while Nursakinah et al. (2012) have conducted a study on the leaf and fresh fruit [5,14]. For the leaf, the proximate compositions from both studies exhibited small differences. As for the fruits, the proximate composition of both studies is significantly different due to differences in fruit conditions used. Both studies have reported the presence of minerals such as calcium, phosphorus, and potassium. Further, in 2014, Kasum and Mirfat reported the proximate composition of freeze-dried *G. atroviridis* fruits from various locations [15]. However, even when the same drying method is used, the moisture content of the fruit powder varies significantly from 0.24 to 23.21%. As those fruits were obtained from different locations, the composition of the fruit, such as sugar and carbohydrates, might affect the hygroscopicity of the fruit powder. In addition, the maturity of the fruit might be different. This hypothesis is supported by Karo-Karo et al. (2019), who reported that ripe dried fruit has a higher moisture content compared to dried unripe fruit [16].

Although the studies on the proximate composition of *G. atroviridis* are very limited, there are numerous studies on the phytochemical content of the plants. Various plant parts such as fruit, root, and stem bark have been studied to investigate the phytochemical components and health benefits. A list of compounds found to be present in the various parts of *G. atroviridis* are documented in Table 2. In addition, the major biological activities of each of the compounds inferred from the previous studies are also presented in Table 2.

**Figure 3:**
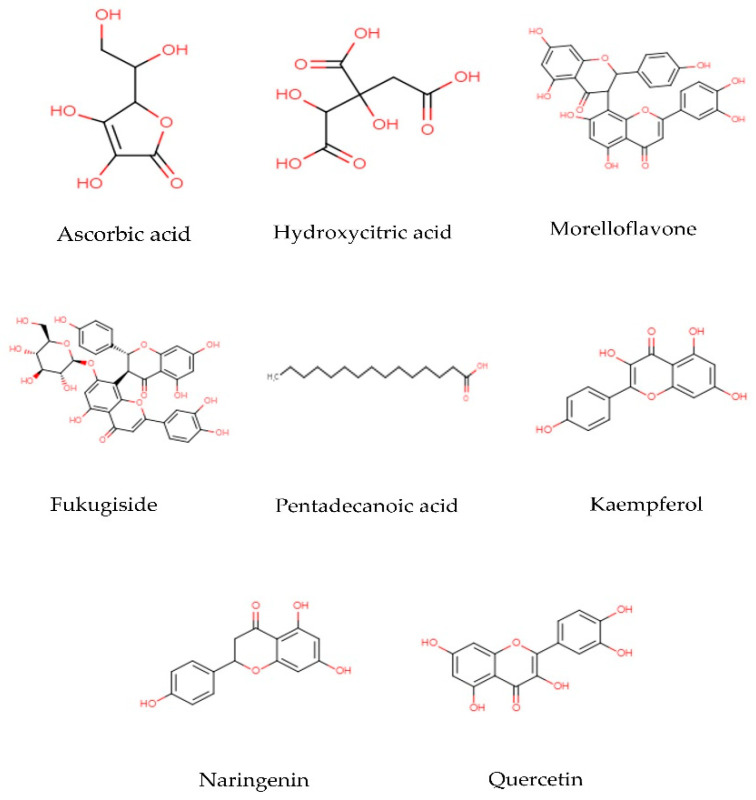
Two dimensional structures of biological active compounds found in the *G. atroviridis*.

It has been seen that the main components found in *G. atroviridis* extracts are organic acids and flavonoids. The structures of some of the biologically active compounds are illustrated in Figure 3. Organic acids are organic compounds with acidic properties that are mostly contributed by their carboxyl groups –COOH, while flavonoids are a group of natural substances with variable phenolic structures. These compounds are among the naturally occurring components that are present in a broad variety of foods, including fruits, vegetables, spices, and medicinal plants. They are well known to have potent antimicrobial, antioxidant, and anti-inflammatory activities. For example, citric acid in particular seems to have a key role in antibacterial action and skin health [57]. The presence of xanthone can also contribute to the therapeutic effect of this plant extract. Xanthones are simple three-membered ring compounds that have very diverse biological profiles depending on their various structures, such as antihypertensive, antioxidative, antithrombotic, and anticancer activities [58]. According to a recent study, xanthones have the ability to inhibit α-glucosidase activity [59]. Although previous studies have reported the presence of succinic acid, garcinol, isogarcinol, and camboginol in *G. atroviridis* [18,35,36], all these reports refer to all Garcinia species in general. No specific study has reported the presence of these compounds in *G. atroviridis*.

## 6. Biological Activities of *G. atroviridis* and its Constituents

### 6.1. Antimicrobial

Pathogenic microorganisms have been a threat to humanity from its inception. Infectious diseases, especially in developing countries, are the leading cause of morbidity and mortality in the general population [60]. For the past two decades, pharmaceutical companies and different research groups have been conducting studies using natural products to develop novel antimicrobial drugs that can work more efficiently against microorganisms, especially multi-resistant drug pathogens [61].

The antimicrobial potential of *G. atroviridis* has been demonstrated by a series of investigations conducted by several research groups. In 2000, Mackeen et al. conducted a study to evaluate the antimicrobial potential of the fruits, roots, leaves, stems, and trunk bark of *G. atroviridis* [11]. They prepared methanol extracts of all plant parts of *G. atroviridis* and determined their antimicrobial effect using three phytopathogenic fungi, i.e., *Cladosporium herbarum*, *Fusarium moniliforme*, and *Alternaria* sp., and seven microbial strains, which were *Bacillus subtilis B28*, *B. subtilis B29*, methicillin-resistant *Staphylococcus aureus* (MRSA), *Escherichia coli, Pseudomonas aeruginosa* UI 60690, *Candida albicans* (yeast), and *Aspergillus ochraceous* ATCC 398 (fungus) [11]. Results revealed that among all the prepared extracts, the root extract exhibited the highest inhibition against all the test bacteria at a minimum inhibitory dosage (MID) of 15.6 µg/disc [11]. It was observed that *G. atroviridis* had weak antifungal potential, and only fruit and leaf crude extracts of *G. atroviridis* exhibited a notable antifungal activity against *C. herbarum* (MID: 100 µg/spot and 400 µg/spot, respectively) compared to the other extract types [11]. Later, Mackeen et al. (2002) performed another study and isolated two garcinia acid derivatives, which were 2-(butoxycarbonylmethyl)-3-butoxycarbonyl-2-hydroxy-3-propanolide and 1′,1″-dibutyl methyl hydroxycitrate from the fruit of *G. atroviridis*. Both phytochemicals exhibited selective antifungal effects comparable to cycloheximide (MID: 0.5 µg/spot) only against *C. albicans* at MID 0.4 and 0.8 µg/spot, respectively, and were active against selected bacterial strains, which were *E. coli*, MRSA, *B. subtilis*, and *P. aeruginosa* [49]. A similar study was performed by Permana et al. (2001) and isolated two novel prenylated phytochemicals, namely, depsidone atrovirisidone and benzoquinone atrovirinone from the roots of the Garcinia plant. Their research findings revealed that both compounds are mildly inhibitory against *S. aureus* and *B. cereus* [31].

Later, a study carried out by Basri et al. (2005) prepared ethanol and ethyl acetate extracts to evaluate the antimicrobial activity of *G. atroviridis* fruits. Their results revealed that both extracts are active against all seven test bacterial strains (four Gram-negative and three Gram-positive) and two yeast strains. However, the ethyl acetate extract showed the strongest inhibitory effect against two Gram-negative bacteria (*S. epidermidis* and *S. aureus*) compared to the positive control drug (gentamycin) [62]. A recent research study performed by Tan et al. (2013) obtained the volatile constituent of *G. atroviridis* fruit and evaluated the antibacterial activities using five bacterial strains, three of which were Gram-negative (*Salmonella typhimurium*, *E. coli*, and *P. stutzeri*) and two Gram-positive (*S. aureus* and *B. subtilis*) [8]. In the fruit of *G. atroviridis*, they isolated three highly abundant compounds, which were α-humulene, β-caryophyllene alcohol, and (−)-β–caryophyllene that exhibited stronger antibacterial potential against tested Gram-positive bacterial strains compared to the Gram-negative bacteria [8]. A more recent research study conducted by Thongkham et al. (2021) also evaluated the antimicrobial potential using an ethanol extract of *G. atroviridis* fruit [63]. Their findings revealed that the ethanolic fruit extract of *G. atroviridis* showed antimicrobial activity against *E. coli* TISTR 073, *Streptococcus agalactiae* ATCC 27956, *B. subtilis* DMST 3763, *S. intermedius* DMST 5024, *S. epidermidis* DMST 12853, and *S. aureus* DMST 4745; and two strains of yeast, *C. albicans* ATCC 10, 231, and *Malassezia pachydermatis* [63].

Unlike the previous studies, Suwanmanee et al. (2014) demonstrated that *G. atroviridis* fruit extract has antifungal activity when tested against several strains of fungi, which were *A. niger*, *Microsporum gypseum*, *C. albicans, Microsporum canis*, *Saccharomyces cerevisiae*, *Epidermophyton floccosum, Trichophyton mentagrophytes*, *Trichophyton tonsurans*, and *Penicillium* spp. [64]. All these studies revealed remarkable antimicrobial potential in various parts, different extracts, or constituents of *G. atroviridis*. Generally, drugs derived from natural products use different mechanisms of action, such as biofilm formation and membrane disruption; and the inhibition of cell envelope synthesis, nucleic acid synthesis, the electron transport chain, bacterial toxins, and bacterial efflux pumps, which are useful in improving antibacterial therapy [65]. Despite these studies, the exact mechanism of action of *G. atroviridis* still needs to be discovered.

### 6.2. Antioxidant

Oxidative stress has been linked to a number of diseases, including cancer, cardiovascular, neurological, and even ageing. A diet high in antioxidants has been claimed to be advantageous to human health. Various approaches have been developed to determine the antioxidant potential of natural products, such as chemical-based and cellular-level-based evaluations [66]. The chemical-based approaches assess a sample’s ability to block the oxidation of a target molecule. These chemical-based assays determine the (a) scavenging activity toward stable free radicals, which are DPPH (1,1-diphenyl-2-picrylhydrazyl) and ABTS (2,2-Azinobis-(3-ethylbenzothiazole-6-sulphonate) radical cation; (b) reduction of metal ions, which are FRAP (ferric reducing antioxidant power) and CUPRAC (Cupric ion-Reducing Antioxidant Capacity) assays; (c) competitive methods (ORAC: oxygen radical absorbance capacity and TRAP: total radical-trapping antioxidant parameter assays); (d) oxidation of low density lipoprotein (LDL); and (e) a more recent and novel method using assays based on nanoparticles. Chemical-based procedures are good for screening as they are low-cost, high-throughput, and produce an index value (expressed in Trolox equivalents) that can be used to compare and sort various natural products. Cellular level assays determine cellular antioxidant activity (CAA), antioxidant enzyme expression levels, and activation or repression of redox transcription factors [66].

To determine the antioxidant potential of *G. atroviridis*, several research studies have been conducted where DPPH, FRAP, FTC (ferric thiocyanate), and TBA (thiobarbituric acid) assays were used (Table 3). The first comprehensive research study conducted by Mackeen et al. (2000) reported that *G. atroviridis* plant parts, which are fruits, leaves, bark, and stems, possess relative antioxidant potential [11]. The FTC and TBA assays used revealed that *G. atroviridis* fruits had high antioxidant potential (60–90% for FTC and 87–93% for TBA) when compared to other plant parts [11]. Nursakinah et al. (2012) used DPPH and FRAP assays to determine antioxidant capacity and found that *G. atroviridis* matured leaves had a higher antioxidant capacity (92.34 percent, 2.47 mmol/L) than young leaves (80.70 percent, 1.90 mmol/L) [14]. Furthermore, the antioxidant activity of young fruits (1.63 mmol/L) was found to be substantially greater than that of matured fruits (1.47 mmol/L) when measured using the FRAP test. The antioxidant potential of these two samples was not significantly different according to the DPPH test. Another recent study performed by Al-Mansoub et al. (2013) prepared aqueous and methanol extracts from leaves, fruit, and stems of *G. atroviridis* and reported that the methanolic extract of the stems exhibited higher antioxidant potential compared to the leaves and fruit (Table 3) [67]. Later, in 2016, Tan et al. (2016) carried out research using the stem bark of *G. atroviridis* to identify antioxidant compounds [52]. They determined the antioxidant capacity using the DPPH assay and found that only 1,3,7-trihydroxyxanthone and quercetin (16.20 and 12.68 μg/mL, respectively) exhibited the highest antioxidant potential among the nine identified compounds [52].

A recent study performed by Chatatikun et al. (2020) determined the antioxidant capacity of *G. atroviridis* fruit pericarps using DPPH and ABTS assays [68]. They prepared an aqueous extract and reported that *G. atroviridis* fruit pericarp is also a good source of antioxidants (Table 3) [68]. More recently, Thongkham et al. (2021) conducted research to determine the antioxidant power of the *G. atroviridis* fruit [63]. They prepared an ethanolic extract of *G. atroviridis* fruit and measured the antioxidant potential by using the DPPH assay, which revealed that the ethanolic fruit extract also exhibited good antioxidant function [63]. Although such findings were reported by various previous studies, it can be concluded that the fruits and leaves of *G. atroviridis* were shown to be high in antioxidants, which can help fight free radicals. All these studies highlighted the remarkable antioxidant potential of different parts of *G. atroviridis,* but researchers should carry out detailed studies to validate the antioxidant potential using both chemical and cell-based assays. In addition, it is also essential to evaluate the highly active antioxidant compounds in the *G. atroviridis* plant.

### 6.3. Anti-Inflammatory

Inflammation is caused by infectious microorganisms such as viruses, bacteria, or fungi invading the body; residing in specific tissues; and/or circulating in the blood. It can also occur as a result of tissue damage, ischemia, malignancy, cell death, and degeneration [69]. Generally, both the innate immune response, which includes mast cells, macrophages, and dendritic cells, and the adaptive immune response, such as B and T cells that produce particular receptors and antibodies, are implicated in the development of inflammation [70]. In response to inflammation, a variety of inflammatory mediators (pro and anti-inflammatory) are synthetized and released. These mediators can be chemokines (e.g., monocyte chemoattractant protein 1), cytokines (e.g., interleukins (ILs), interferons, and tumor necrosis factor (TNF)), eicosanoids (e.g., prostaglandins and leukotrienes), and the potent inflammation-modulating transcription factor: nuclear factor κB (NF-κB) [71]. TNF-α and IL-10 are the most common of the potent pro and anti-inflammatory mediators [72,73]. On the other hand, prostaglandin (PG) E2 is the most studied PG linked to pathological conditions, including inflammatory disorders. Phospholipase A2 produces arachidonic acid from cell membrane phospholipids, which is the first step in the production of PGs (PLA2) [71]. The enzyme cyclooxygenase (COX) then converts arachidonic acid to PGs. The inducible enzyme COX-2 is characterized as the most active during inflammatory processes among the three known COX isoforms (COX-1, COX-2, and COX-3) and is considered the primary target for potential anti-inflammatory candidates [71]. Similarly, nitric oxide synthase (NOS), which creates nitric oxide (NO), is another enzyme linked to inflammatory conditions [24]. Inducible NOS (iNOS), like COX-2, is the most pro-inflammatory NOS isoform [74]. However, long-term inflammation may lead to the progression of chronic inflammation-associated diseases, for instance, cancer, cardiovascular diseases, diabetes, arthritis, colitis, and sepsis [75]. There are several worthwhile natural products that have potent anti-inflammatory potential and are widely used to treat various inflammatory diseases [71].

According to some previous research studies, *G. atroviridis* contains constituents that have anti-inflammatory potential [30]. A benzoquinone, namely, atrovirinone, was previously isolated from *G. atroviridis*. This compound, at inhibitory concentrations (IC_50_) of 4.62 and 9.33 mol/L, downregulates the generation of prostaglandin E2 (PGE2) and nitric oxide, respectively, from IFN-gamma-induced and LPS-induced RAW 264.7 cells and whole blood [76]. Atrovirinone suppressed the production of thromboxane B2 (TXB2) by the cyclooxygenase (COX)-1 and COX-2 pathways at the IC_50_ of 7.41 and 2.10 mol/L, respectively. It can also reduce the production of intracellular reactive oxygen species (ROS) and the release of TNF alpha from RAW 264.7 cells in a dose-dependent manner at the inhibitory concentrations of 5.99 and 11.56 mol/L, respectively [76]. Research studies also found that atrovirinone moderately decreased the lipoxygenase activity. The findings revealed that atrovirinone inhibited the nuclear factor-kappa B (NF-κB) pathway as well as the COX/lipoxygenase enzyme activity, potentially via inhibiting key pro-inflammatory mediators.

**Figure 4:**
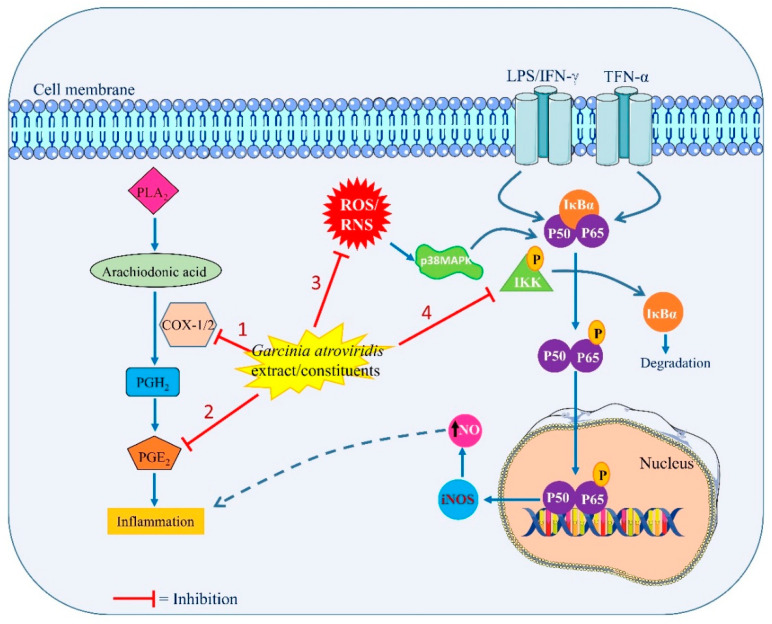
Probable anti-inflammatory mechanisms of action of *G. atroviridis* or its constituents by: (1) inhibiting COX1/2 enzymes activity, (2) affecting the PGE2 production, (3) reducing the ROS/RNS production, and (4) targeting MAPK and NF-κB signaling pathways. PLA2 (Phospholipase A2), COX1/2 (cyclooxygenase 1 and 2), PGH2 (prostaglandin H2), PGE2 (prostaglandin E2), ROS (reactive oxygen species), RNS (reactive nitrogenous species), iNOS (inducible nitric oxide synthase), and MAPK (mitogen-activated protein kinase).

Further research explored that atrovirinone decreased IL-1beta and IL-6 production in a dose-dependent fashion while increasing the secretion of IL-10, an anti-inflammatory cytokine. Atrovirinone also blocked I-kappaB alpha (IκBα) phosphorylation, resulting in a decrease in p65NF-κB nuclear translocation. These results suggest that atrovirinone could be a possible anti-inflammatory medication that targets both the MAPK and NF-κB pathways [5]. These results lend even more credence to the use of *G. atroviridis* as a potent anti-inflammatory natural product. Figure 4 summarizes the probable anti-inflammatory mechanisms of action of *G. atroviridis* or its constituents.

### 6.4. Antihyperlipidemic/Anti-Obesity/Antidiabetic

**Figure 5:**
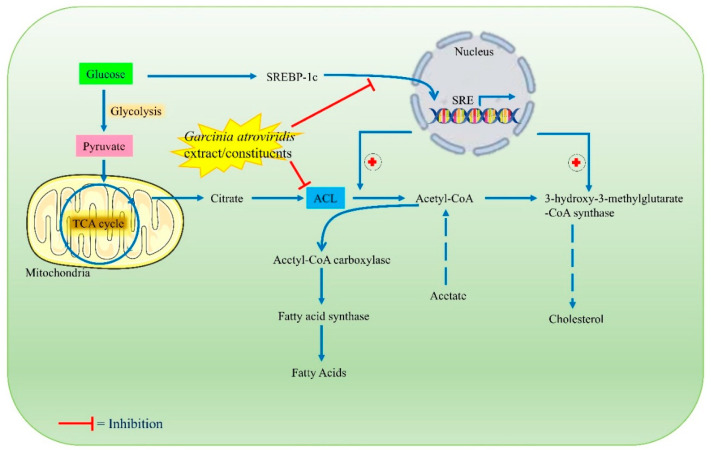
Probable antihyperlipidemic/anti-obesity mechanisms of action of *G. atroviridis* extracts or its constituents.

The *G. atroviridis* fruits are reported to contain fruit acids and saturated fatty acids such as ascorbic acid, tartaric acid, malic acid; and pentadecanoic (15:0), octadecanoic (18:0, stearic acid), nonadecanoic (19:0, nonendocytic acid), and dodecanoic (12:0, lauric acid) acids [77,78]. In addition, it was discovered to contain (−)-hydroxycitric acid (HCA) and flavonoids that have been shown to possess remarkable hypolipidemic effects, promoting weight reduction by reducing lipogenesis and enhancing glycogen formation. In mammals, HCA is an intoxicating metabolic regulator of obesity and lipid disorders. HCA, the main acid found in *G. atroviridis* fruits, has been demonstrated to be a competitive inhibitor of ATP (adenosine 5′-triphosphate) citrate lyase (ACL). This enzyme assists in catalyzing the extramitochondrial cleavage of citrate to oxaloacetate and acetyl coenzyme A (CoA). This activity is assumed to restrict the availability of acetyl CoA, which is essential for the initial stage in the production of fatty acids and cholesterol, as well as lipogenesis during a high lipogenic diet (Figure 5). Moreover, the conducted research on animals revealed that HCA decreased fatty acid synthesis, food intake, and lipogenesis; and induced weight reduction [79].

A group performed research on rats using *G. atroviridis* fruit juices. Daily treatment with 2 mL of 2 percent potassium for two weeks reduced serum cholesterol LDL (low-density lipoprotein) levels from 63 mg/day to 50 mg/day, improved HDL (high-density lipoprotein) concentrations from 35 to 63 mg/day, and decreased body weight. The findings suggest that *G. atroviridis* fruit might be used as a dietary supplement to reduce blood cholesterol levels and lose weight [79]. Another previous research study conducted on poloxamer-407-induced hyperlipidemic rats evaluated the antihyperlipidemic effect of crude methanol and an aqueous extract of six different parts of *G. atroviridis* [80]. They fed the rats with 1000 mg/kg of body weight for three days and observed that the aqueous extract of ripe fruits with seeds among the six different extracts demonstrated a remarkable reduction in cholesterol and triglyceride levels in serum compared to the hyperlipidemic control [80]. A study conducted by Kongchian et al. (2019) evaluated the antihyperlipidemic and anti-obesity potential of *G. atroviridis* fruit extract [81]. They performed both in vitro and in vivo experiments. The in vitro results of the study exhibited that the *G. atroviridis* fruit extracts have significant inhibitory potential against amylase and glucosidase enzymes. In the in vivo study, they administered high-fat diet mice orally with the *G. atroviridis* extract once daily. The results displayed a remarkable reduction in body weight, blood glucose levels, total cholesterol, triglyceride, and LDL cholesterol. A significant increment in HDL cholesterol was also observed. Furthermore, the pathohistology of liver tissues showed a decreased malondialdehyde (MDA), which is a lipid peroxidation marker, indicating a reduction in fat cell deposition [81].

A recent study performed by Susanti et al. (2020) evaluated the lipid modulation effect of *G. atroviridis* fruit extract in 3T3-L1 adipocyte cells and also quantified the HCA amount present in the extract. They observed that a *G. atroviridis* extract significantly affects the expression of C/EBPα (Figure 5). Also, increased adipolysis was noted upon treatment with the *G. atroviridis* fruit extract in a dose-dependent manner [82]. A more recent study conducted by Lim et al. (2020) investigated the anti-obesity potential of a *G. atroviridis* fruit methanolic extract. Upon administration of the *G. atroviridis* extract to obese female rats, a remarkable reduction in weight gain and improved lipid profile was observed, indicating that the methanolic extract also possessed anti-obesity potential [83]. Figure 5 summarizes probable antihyperlipidemic/anti-obesity mechanisms of action of *G. atroviridis* or its constituents.

### 6.5. Anticancer

**Figure 6:**
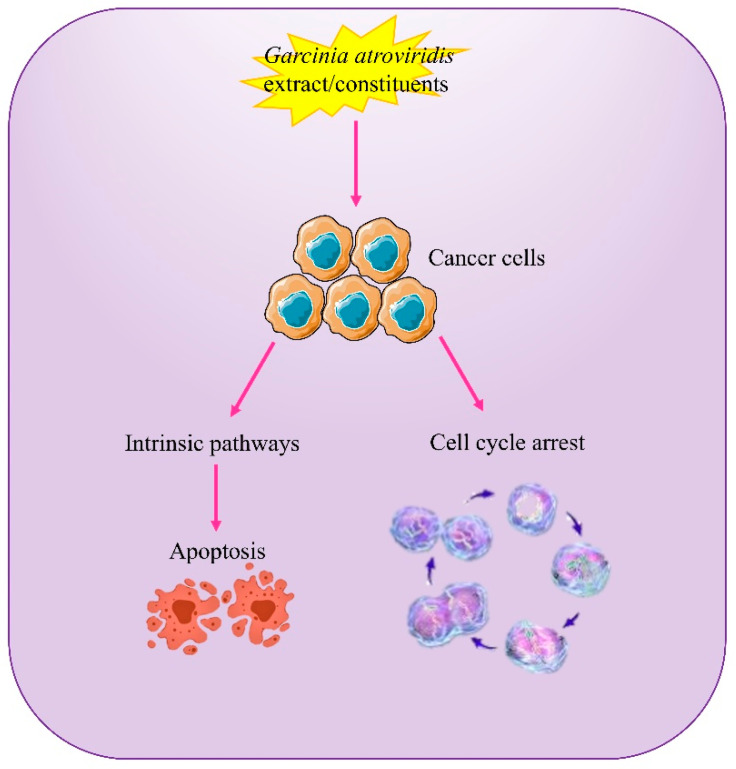
Schematic illustration of the probable anticancer mechanisms of action of *G. atroviridis* extracts or its constituents.

An anticancer potential of the compounds was also discovered in *G. atroviridis*. Two prenylated substances isolated from the roots of *G. atroviridis*, the benzoquinone atrovirinone and the depsidone atrovirisidone, demonstrated pharmacological action [31]. Subsequently, a research group discovered that the phytochemical, atrovirisidone, exhibited a cytotoxic effect against HeLa cells. Another compound isolated from the roots of *G. atroviridis*, atrovirisidone B, which is a prenylated depsidone, together with naringenin and 3,8′′-binaringenin, demonstrated cytotoxicity against human breast (MCF-7), lung (H-460), and prostate (DU-145) cancer cells [32]. In contrast to its anticancerous activity, it has also been seen that the extract of *G. atroviridis* was one of 43 plant species that increased the in vitro cell survival of a human leukemia cell-line HL60 at a concentration of 20 mg/mL by more than 50% when exposed to 9.6 J/cm2 of wide spectrum light [84]. The findings suggested that the plant extract could also be useful in photodynamic treatment. Figure 6 summarizes the probable anticancer mechanisms of action of *G. atroviridis* or its constituents.

## 7. Future Perspective

*G. atroviridis* has a long history of curing a variety of diseases, but only a few pharmacological studies have been done to date to support these claims. The data on extraction methods is limited; thereby, further research may be conducted utilizing several extraction techniques, including microwave extraction, etc., for the isolation and identification of active phytoconstituents. The presence of a diverse variety of phytochemicals in various parts indicates that *G. atroviridis* could pave the way for the development of promising drugs that work efficiently. The plant contains a variety of phytochemicals; however, it is important to isolate the active compounds using the appropriate chromatographic procedures. *G. atroviridis* seems to possess a great potential for in-depth research into a variety of biological functions. Therefore, it is important to explore its maximum therapeutic potential in order to enable its successful application in medicinal and pharmaceutical fields. Currently, the authors are involved in investigating the antidiabetic and anti-obesity potential of plants, including *G. atroviridis*, with the aim of the identification and isolation of bioactive phytoconstituents.

## 8. Conclusions

All the previous research studies tried to uncover the therapeutic potential of *G. atroviridis*. It was found that the composition of this plant extract varies depending on the part of the plant as well as the location where it grows. There are 38 compounds that have been identified and present in the various parts of this plant in the form of organic acids, flavonoids, and many more. Although this plant has been used as a traditional medicinal ingredient for a long time, current studies have proven its goodness to human health by having antimicrobial, antioxidant, and anti-inflammatory capabilities. Studies have demonstrated that *G. atroviridis* extracts or constituents exert their antioxidant potential by scavenging free radicals or by inhibiting the various molecular targets involved in oxidative stress production. It works against inflammation by inhibiting PGE2 and NO from macrophages or by downregulating NF-κB/COX pathways. In addition, *G. atroviridis* extracts or constituents can act as an antihyperlipidemic by inhibiting the activity of ATP citrate lyase, which is required for the cleavage of citrate to oxaloacetate and restricting the availability of acetyl coenzyme A (CoA). *G. atroviridis* can also alleviate the risk of different cancer types, although many in vitro and in vivo research studies still need to be conducted further to uncover the underlying mechanisms of action. Despite this, the conducted work provides a wide range of therapeutic data on *G. atroviridis* for the treatment or prevention of a series of chronic disorders.

## Figures and Tables

**Table 1:** Proximate composition of *G. atroviridis*.

Part	Moisture	Fat (%)	Protein (%)	Carbohydrate (%)	Ash (%)	Mineral	Energy (Kcal)	Source
young leafy shoot	79.1	0.4	1.8	15.5	0.6	Ca, P, Fe, Na, K, carotenes, niacin, vitamin A, B1, B2, and C	73	[5]
dried fruit	30.3	1.3	2.7	51.9	1.6	Ca, P, Fe, Na, K, carotenes, niacin, vitamin A, B1, B2, and C	-	
leaf	81.03	0.12	2.16	15.98	0.72	Ca, P, K, Al, S, Br	73.64	[14]
fresh fruit	90.52	0.22	0.46	8.64	0.15	Ca, P, K, Al, S, Br	38.38	
fresh fruit	0.24–23.21	0.35–0.81	1.06–2.42	76.86–96.03	1.36–2.06	-	303–397	[15]
dried unripe fruit	5.43	-	-	-	1.83	-	-	[16]
dried ripe fruit	6.19	-	-	-	1.82	-	-	

**Table 2:** Phytochemicals reported in various parts of *G. atroviridis* with their main biological activities.

Compound Name	Class	Part Used	Biological Activities	References
ascorbic acid	organic acid	fruit	antioxidant, anticancer, epigenetic regulator, beverages	[17,18]
citric acid	organic acid	fruit	antioxidant,	[18,19]
malic acid	organic acid	fruit	beverages, chelating agent, buffering	[18,20]
succinic acid	organic acid	fruit	food additive	[18,21]
tartaric acid	organic acid	fruit	food additive	[18]
hydroxycitric acid	organic acid	fruit	anticancer, anti-obesity, anti-inflammatory	[22,23,24]
pentadecanoic acid	organic acid	fruit	antimicrobial, anticancer, antihyperglycemic, anti-inflammatory	[18,25,26,27,28]
nonadecanoic acid	organic acid	fruit	-	[18]
dodecanoic acid	organic acid	fruit	-	[18]
14-cis-docosenoic acid	organic acid	root	-	[29]
atroviridin	pyranoxanthones	stem bark	-	[7]
benzoquinone atrovirinone	ubiquinones	root	anti-inflammatory	[30,31]
atrovirisidone	depsidones	root	-	[31]
atrovirisidone B	depsidones	root	anticancer	[32]
garcineflavonol A	flavonoid	stem bark	-	[33]
garcineflavanone A	flavonoid	stem bark	-	[33]
garcinol	terpenoid	fruit	antioxidant, anticancer, anti-inflammatory, anti-glycation, antiulcer, antibacterial	[18,34,35,36]
α-humulene	sesquiterpenoid	fruit	antitumor, anti-inflammatory, antibacterial	[8,37,38]
isogarcinol	terpenoid	fruit	antioxidant, anticancer, anti-inflammatory, antibacterial	[18,36]
β-caryophyllene alcohol	sesquiterpenoid	fruit		[8]
(−)-β-caryophyllene	sesquiterpenoid	fruit	antioxidant, antitumor, anti-inflammatory, antibacterial, anticancer, anticonvulsant	[8,39,40,41]
camboginol	prenyltated xanthone	fruit	antioxidant	[18,42]
naringenin	flavonoid	root	antioxidant, anticancer, antidiabetic, anti-inflammatory, antiproliferative, antimutagenic, antiatherogenic	[32,43,44]
3,8″-binaringenin	flavonoid	root		[32]
morelloflavone	flavonoid	root	antiproliferative, anti-inflammatory, antihyperlipidemic, anticancer	[29,45,46,47,48]
4-methylhydroatrovirinone	benzenoid	root	-	[29]
1′,1″-dibutyl methyl hydroxycitrate	organic acid	fruit	-	[49]
2-(butoxycarbonylmethyl)-3-butoxycarbonyl-2-hydroxy-3-propanolide	organic acid	fruit	-	[49]
fukugiside	flavonoid	root	antioxidant, antibacterial	[29,50,51]
garcinexanthone G	xanthone	stem bark	antioxidant	[52]
stigmasta-5,22-dien-3β-ol	sterol	stem bark	antioxidant	[52]
stigmasta-5,22-dien-3-O-β-glucopyranoside	steroid saponin	stem bark	antioxidant	[52]
3β-acetoxy-11α,12α-epoxyoleanan-28,13β-olide	triterpenoids	stem bark	antioxidant	[52]
2,6-dimethoxy-p-benzoquinone	benzoquinone	stem bark	antioxidant, antibacterial	[52,53]
1,3,5-trihydroxy2-methoxyxanthone	xanthone	stem bark	antioxidant	[52]
1,3,7-trihydroxyxanthone	xanthone	stem bark	antioxidant, neurotropic factor	[52,54]
kaempferol	flavonoid	stem bark	antioxidant, anti-inflammatory, antiatherogenic, hepatoprotective, cardioprotective, neuroprotective anticancer	[52,55]
quercetin	flavonoid	stem bark	antioxidant, antidiabetic, anti-inflammatory, antimicrobial, anti-Alzheimer’s, antiarthritic, cardioprotective	[52,56]

**Table 3:** Antioxidant activities of various extracts of *G. atroviridis*.

Plant Part	Extract Type	Antioxidant Test	Results	Reference
fruit, leaf, bark, and stem,	methanol	FTC and TBA	-fruits showed high antioxidant potential compared to other plant parts	[11]
matured and young leaf and fruit	aqueous (distilled hot water)	DPPH and FRAP	-matured leaves and young fruits had higher antioxidant capacity	[14]
leaf, fruit, and stem	aqueous and methanol	DPPH, ABTS, and FRAP	-all methanol extracts of all parts showed comparatively better potential than aqueous extracts -stem exhibited higher antioxidant potential among methanol part extracts	[67]
stem bark	chloroform, hexane, dichloromethane, and methanol	DPPH	-among all the nine identified compounds from the fractions, 1,3,7-trihydroxyxanthone exhibited the highest antioxidant potential	[52]
fruit pericarp	aqueous (hot water)	DPPH and ABTS	-fruit pericarp also has good antioxidant potential	[68]
fruit	ethanol	DPPH	-ethanol fruit extract has good antioxidant property	[63]

## Data Availability

Not applicable.
